# Thyroid Hormone Dynamics and DIO2 Variants in Schizophrenia: Exploring Genetic Links to Neuroendocrine Imbalance

**DOI:** 10.1111/jcmm.70694

**Published:** 2025-06-30

**Authors:** Gokce Akan, Ismael Chatita Adolf, Adil Colak, Seda Acar, Fatih Oncu, Dogan Yesilbursa, Solmaz Turkcan, Fatmahan Atalar, Sema Bilgic Gazioglu

**Affiliations:** ^1^ Biochemistry Department, MUHAS Genetics Laboratory, School of Medicine Muhimbili University of Health and Allied Sciences Dar es Salaam Tanzania; ^2^ DESAM Research Institute Near East University Mersin 10 Türkiye; ^3^ Department of Biomedical Engineering, Biomedical Engineering Institute Bogazici University Istanbul Türkiye; ^4^ Department of Bioengineering Yildiz Technical University Istanbul Türkiye; ^5^ Psychiatry Clinics Turkish Ministry of Health Bakirkoy Research and Training Hospital for Psychiatry, Neurology and Neurosurgery Istanbul Türkiye; ^6^ Rare Diseases Research Laboratory, Istanbul Medical Faculty Istanbul University Istanbul Türkiye; ^7^ Department of Rare Diseases, Child Health Institute Istanbul University Istanbul Türkiye; ^8^ Department of Immunology, Institute of Experimental Medicine Istanbul University Istanbul Türkiye; ^9^ Department of Microbiology, School of Dentistry Istanbul Nisantasi University Istanbul Türkiye

**Keywords:** case‐control study, deiodinase enzyme II, DIO2, genotyping, polymorphism, schizophrenia, thyroid hormone

## Abstract

Thyroid hormone modulates multiple neurotransmitter systems, including dopaminergic, serotonergic, glutamatergic and GABAergic pathways, which are implicated in schizophrenia (SCH) pathophysiology. The Type II deiodinase (DIO2) enzyme plays a critical role in thyroid metabolism, converting thyroxine (T4) into the biologically active triiodothyronine (T3). This study aimed to investigate the potential association between DIO2 gene polymorphisms, Thr92Ala and ORFa‐Gly3Asp, with serum levels of free triiodothyronine (fT3), free thyroxine (fT4) and thyroid‐stimulating hormone (TSH) in SCH susceptibility and symptomatology. The cohort included 582 unrelated patients diagnosed with SCH and 603 healthy controls. Genotyping of Thr92Ala and ORFa‐Gly3Asp single nucleotide polymorphisms (SNPs) of the DIO2 gene was conducted along with serum measurements of TSH, fT4 and fT3 levels. The genotype distribution of Thr92Ala and ORFa‐Gly3Asp genotypes differed significantly between SCH group and the controls (*p* < 0.001). Furthermore, patients with SCH exhibited significantly lower levels of fT3 (*p* < 0.001) and TSH (*p* < 0.001) compared with controls. Notably, the Thr92Ala genotypes displayed a significant association with altered fT3 and TSH levels in SCH patients (*p* < 0.05, respectively). This study identified a significant association between DIO2 polymorphisms and decreased levels of fT3 and TSH in Turkish patients with SCH. Given the impact of thyroid hormones on neurotransmitter systems involved in SCH, these results highlight the potential for thyroid hormone modulation as a therapeutic avenue. Further research could lead to more personalised treatment strategies, particularly for patients with genetic predispositions to altered thyroid hormone metabolism, improving clinical outcomes and offering new approaches to managing symptoms in schizophrenia.

**Abbreviations**
*χ*2: chi‐square
AD: Alzheimer's disease
Ala: alanine
ANOVA: assumptions of analysis of variance
BAT: brown adipose tissue
BD: bipolar disorder
BDI‐II: Beck Depression Inventory Scale II
BRSHH: Bakırkoy Mazhar Osman Mental Health and Neurological Diseases Education and Research Hospital
CNS: central nervous system
CTR: controls
DIO1: deiodinase enzymes type 1
DIO2: deiodinase enzymes type 2
DIO3: deiodinase enzymes type 3
DSM‐IV: Diagnostic and Statistical Manual of Mental Disorders‐Fourth Edition
fT3: free triiodothyronine
fT4: free thyroxine
HWE: Hardy–Weinberg equilibrium
MCT8: thyroid hormone transporter monocarboxylate transporter‐8
MR: mental retardation
PBMCs: peripheral blood mononuclear cells
PCR‐RFLP: polymerase chain reaction restriction fragment length polymorphism
SCH: schizophrenia
SCID‐I: Structured Clinical Interview
SERT‐T3: sertraline combined with T3
SNP: single nucleotide polymorphism
T3: 3,5,3′‐triiodothyronine
T4: 3,3′,5,5′‐triiodothyronine
TH: thyroid hormone
Thr: threonine
TSH: thyroid‐stimulating hormone
WMA: World Medical Association

## 1 Introduction

Schizophrenia (SCH) is a complex psychiatric disorder affecting approximately 1.5% of the global population, characterised by considerable clinical heterogeneity that spans across all socioeconomic strata, suggesting multifactorial aetiology [1]. This heterogeneity, along with SCH's multifactorial aetiology, poses significant challenges in pinpointing definitive genetic markers. The current understanding of SCH pathogenesis is guided by two primary genetic hypotheses: polygenic inheritance and variably penetrant de novo mutations in a subset of individuals [2]. Although certain chromosomal loci have been identified in SCH, conclusive identification of causative mutations or disease‐associated variants remains limited [3]. Identification of novel susceptibility genes holds promise for identifying at‐risk individuals and may inform more targeted therapeutic strategies.

Research findings have consistently demonstrated a higher prevalence of SCH among individuals with hypothyroidism compared to those with euthyroid status in the general population [4]. Thyroid hormone (TH) plays a pivotal role in the intricate processes of brain and nervous system development, encompassing neurogenesis and terminal brain differentiation [5]. Recent advancements in the field have underscored the critical importance of TH signalling pathways in regulating key neurobiological mechanisms implicated in SCH pathophysiology. These insights into the thyroid–neuroendocrine axis have highlighted the complex interplay between TH function and the aetiology of SCH, potentially paving the way for novel therapeutic interventions targeting this axis. Many genes modulated by THs exhibit expression within the central nervous system. The regulation of TH‐responsive genes involves dynamic interactions among thyroid hormone receptors, chromatin architecture, DNA sequences and epigenetic modifications. The important role of THs in brain development is underscored by their multifaceted impacts on neuronal differentiation, signalling cascades, myelinogenesis, neuronal cell proliferation and maturation, as well as synaptic formation and transmission [6]. Perturbations in the thyroid system have been implicated in the pathogenesis of diverse psychiatric disorders T3 (3,5,3′‐triiodothyronine) and T4 (3,3′,5,5′‐triiodothyronine) represent the principal hormones of the human thyroid axis, orchestrating a myriad of neurobiological processes critical for central nervous system (CNS) function and development [7].

Three distinct types of iodothyronine deiodinase enzymes, namely Type 1 (DIO1), Type 2 (DIO2) and Type 3 (DIO3), are key regulators of TH metabolism. Of these, DIO2 is particularly significant for the conversion of the prohormone thyroxine; T4 into its biologically active form, triiodothyronine; T3, or its inactive metabolite, reverse T3, within the brain. DIO2 also regulates intracellular T3 concentrations in brown adipose tissue (BAT) and the pituitary gland, significantly influencing systemic TH homeostasis. Its role in T4 to T3 conversion within the brain is critical for ensuring adequate T3 levels during critical developmental stages [8, 9].

The DIO2 gene is located on chromosome 14q24.3 and spans approximately 15 kb and exhibits multiple polymorphic variants, including the clinically significant Thr92Ala polymorphism [10, 11]. This single nucleotide polymorphism (SNP) substitutes threonine (Thr) with alanine (Ala) at codon 92, potentially altering enzyme function [12]. The role of DIO2 in TH regulation within the brain makes it a relevant candidate for investigation in SCH, particularly given the enzyme's broader expression in diverse tissues including the pituitary gland, brain, reproductive organs and BAT where it influences systemic TH homeostasis [13].

The Thr92Ala‐DIO2 polymorphism has been associated with various health conditions such as hypertension, Type 2 diabetes, mental disorders, bone metabolism disturbances and autoimmune thyroid diseases [14, 15]. In SCH, patients frequently exhibit an accumulation of metabolically active T3 in peripheral blood, attributed to enhanced T4 degradation in peripheral tissues [9, 16, 17]. Animal models with altered DIO2 expression support a role for this enzyme in cognitive function including memory and verbal skills [15]. Interestingly, research with DIO2 knockout mouse models has shown that male knockout mice exhibited mild growth retardation and elevated serum T4 and thyroid‐stimulating hormone (TSH) levels, despite unchanged T3 levels suggesting potential compensatory mechanisms [18].

Given the pivotal role of TH in brain development and the regulation of its active form within the brain by DIO2, limited research has explored the potential relationship between allelic variations of the DIO2 gene and mental disorders. The Thr92 residue of DIO2 is typically found localised in the endoplasmic reticulum of cells, whereas the Ala92 variant accumulates in the Golgi apparatus, a shift associated with oxidative stress, disruption of mitochondrial function and apoptotic changes [19]. Previous investigations have revealed distinct transcriptomic profiles in carriers of the Thr92Ala‐DIO2 polymorphism and HEK‐293 cells expressing Ala92‐DIO2 variant. These profiles are characterised by differential expression of genes implicated in central nervous system diseases, ubiquitination, mitochondrial dysfunction, inflammation, DNA repair, apoptosis and growth factor signaling [20]. Subsequently, racial disparities in DIO2‐related neurodegenerative diseases have been investigated and particularly individuals of African American descent with the Thr92Ala‐DIO2 variant were shown to exhibit increased susceptibility to Alzheimer's disease (AD), dementia or cognitive decline without dementia, compared with their counterparts of European American descent [20]. Recent investigations have explored the potential link between bipolar disorder (BD) and mental retardation with two genetic variants of the DIO2 gene; Thr92Ala and ORFa‐Gly3Asp [9, 11, 12, 14]. Notably, while He et al. found significant associations between these genetic variations and BD, two separate studies by Guo et al. and Zhang et al. reported no significant associations with mental retardation [9, 11, 14].

The primary aim of this study was to assess the potential impact of the Thr92Ala and ORFa‐Gly3Asp variants in DIO2 gene on SCH susceptibility, with a focus on the interaction between these polymorphisms and thyroid hormone levels. Specifically, we assessed serum levels of free triiodothyronine (fT3), free thyroxine (fT4) and TSH to investigate the complex relationship between genetic factors, TH function and SCH. Our findings may offer novel insights into thyroid‐related biomarkers for SCH risk assessment and therapeutic potential.

## 2 Materials and Methods

### 2.1 Subjects

This study included 582 unrelated patients (430 men and 152 women; mean age 37.26 ± 8.94) diagnosed with SCH, admitted to Bakırkoy Mazhar Osman Mental Health and Neurological Diseases Education and Research Hospital (BRSHH). The diagnosis of SCH was established by two independent psychiatrists using patient interviews conducted with the Structured Clinical Interview for DSM‐IV (SCID‐I), in accordance with the Diagnostic and Statistical Manual of Mental Disorders‐Fourth Edition (DSM‐IV) [21]. Patients with substance‐induced psychotic disorders, psychosis secondary to a general medical condition, and comorbid psychiatric disorders were excluded. The patients were undergoing treatment with antipsychotic medications: Olanzapine (41.6%), Olanzapine atypical (22.8%), typical (4%) and other combinations (31.7%). The control group consisted of 603 healthy individuals (414 men and 189 women; mean age 37.20 ± 9.03) matched by sex, randomly recruited from volunteer blood donors. To ensure mental health, controls completed the Beck Depression Inventory Scale II (BDI‐II) to screen out depressive symptoms as a confounding factor, and those with psychiatric or neurological disorders were excluded. This study was approved by the Research Ethics Committee of BRSHH according to the WMA Declaration of Helsinki. Written and signed informed consent was obtained from all subjects and/or their legal guardians.

### 2.2 Hormone Measurement

The serum levels of TSH, fT4 and fT3 were measured using the electrochemiluminescence immunoassay (ECLIA) to maintain high sensitivity and specificity. The normal reference ranges for these hormones were set as follows: 0.27–4.2 IU/mL for TSH, 0.93–1.9 ng/dL for fT4 and 1.8–4.3 pg/mL for fT3.

### 2.3 Genotyping Analysis

Genomic DNA was extracted from peripheral blood mononuclear cells (PBMCs) obtained from all study participants using the MagNaPure Compact Nucleic Acid Isolation Kit (Roche Applied Science, Germany), followed by qualitative and quantitative analysis using Picodrop (Picodrop, Saffron Walden, UK). For the genotyping analysis of DIO2 polymorphisms, the polymerase chain reaction–restriction fragment length polymorphism (PCR‐RFLP) method was conducted. For the genotyping analysis of DIO2 polymorphisms, two distinct PCR assays were conducted. Firstly, a 263‐bp fragment encompassing the Thr92Ala polymorphism was amplified utilising forward primer 5′‐AATGTAGACCAGCAGGGAAGT‐3′ and reverse primer 5′‐AGGTGAAATTGGGTGAGGAT‐3′. Additionally, a 145‐bp fragment covering the ORFa‐Gly3Asp polymorphism was amplified using forward primer 5′‐AAAGCTGGCGTACTCGTC‐3′ and reverse primer 5′‐AAAGAGCATAGAGACAATGAAAG‐3′, as previously described [9].

The PCR conditions involved an initial denaturation step at 95°C for 5 min, followed by 35 cycles comprising denaturation at 95°C for 45 s, annealing at 53.8°C for 30 s, and extension at 72°C for 30 s for Thr92Ala and denaturation at 95°C for 5 min, followed by 35 cycles comprising denaturation at 95°C for 45 s, annealing at 51°C for 30 s and extension at 72°C for 30 s for ORFa‐Gly3Asp. A final extension step at 72°C for 10 min concluded the PCR process. Subsequently, the amplified PCR products underwent enzymatic digestion using the restriction endonucleases *RsaI* (Fermentas) for the 263‐bp fragment and *CviKI‐1* (Fermentas) for the 145‐bp fragment, respectively, at 37°C overnight. The digested PCR products were then separated and visualised on 2% agarose gels for PCR fragments and 3% agarose gels for restriction fragments under UV light.

### 2.4 Statistical Analysis

Statistical analyses were performed using the SPSS software package (revision 15.0, SPSS Inc., Chicago, IL). Differences between normally and non‐normally distributed continuous variables were assessed using Student's *t*‐test and Mann–Whitney *U*‐test, respectively. Mean values and standard deviations were computed for continuous variables. Genotype and allele frequencies were compared employing the chi‐squared (*χ* ^2^) test. The genotypic distributions at each SNP were assessed for Hardy–Weinberg equilibrium (HWE) using the *χ* ^2^ test. Relative risks with 95% confidence intervals were estimated as odds ratios. To compare differences between groups, data underwent logarithmic transformation to meet the assumptions of analysis of variance (ANOVA), followed by one‐way ANOVA with Tukey's post hoc analysis. Statistical significance was defined as *p* < 0.05.

## 3 Results

The distributions of genotype and allele frequencies for the Thr92Ala and ORFa‐Gly3Asp polymorphism in the DIO2 gene among SCH patients (SCH) and controls (CTR) are presented in Table 1. For the ORFa‐Gly3Asp polymorphism, the frequencies in the SCH group were 35.1% (CC), 43.5% (CT) and 21.5% (TT), while in the CTR group, the frequencies were 53.6%, 34.2% and 12.3%, respectively. Similarly, the Thr92Ala polymorphism frequencies in the SCH group were 35.7% (TT), 35.6% (TC) and 28.7% (CC) compared with 60.9%, 27.9% and 11.3% in the CTR group. Significant differences in both polymorphisms were observed between SCH and CTR groups (*p* < 0.05). The risk allele frequencies of both SNPs were also statistically significant in the SCH group compared with the CTR group (*p* < 0.05), with risk allele frequencies markedly elevated in the SCH group.

**TABLE 1:** Genotypic and allelic frequency distributions of SNPs in the subgroups.

SNP	Genotypic frequencies *n* (%)		*p* ^a^	Allelic frequencies			*χ* ^2^	OR/CI (95%)	*p* ^a^
Genotype	SCH (*n* = 582)	Controls (*n* = 603)		Allele	SCH (*n* = 582)	Controls (*n* = 603)			
rs12885300 (ORFa‐Gly3Asp)									
CC	204 (35.1)	323 (53.6)							
CT	253 (43.5)	206 (34.2)	0.001	C/T	0.57/0.43	0.71/0.29	48.58	1.82/1.54–2.16	0.001
TT	125 (21.5)	74 (12.3)							
rs225014 (Thr92Ala)									
TT	208 (35.7)	367 (60.9)							
TC	207 (35.9)	168 (27.9)	0.001	T/C	0.54/0.46	0.75/0.25	116.8	2.57/2.16–3.06	0.001
CC	167 (28.7)	68 (11.3)							

Abbreviations: CI, confidence interval; *n*, number; OR, odd ratio; SCH, schizophrenia.

^a^ p‐Values of the genotypic and allelic frequency distributions of polymorphisms between the groups were compared using *χ* ^2^ and HWE test. In all cases, differences were considered significant at *p* < 0.05.

Within the SCH group, the distribution of the Thr92Ala genotypes also varied by sex (Table 2). Among male SCH patients, the TT, TC and CC genotype frequencies were 37.2%, 33.3% and 29.5%, respectively, versus 60.4%, 28.0% and 11.6% in male controls. In female SCH patients, the genotype frequencies were 31.6% (TT), 42.1% (TC) and 26.3% (CC), compared with 61.9%, 27.5% and 10.6% in female groups (*p* < 0.001). These findings indicate statistically significant differences between SCH and control groups for both males and females (*p* < 0.001).

**TABLE 2:** Gender distribution of the Thr92Ala polymorphism in patients with SCH.

Gender	Thr92Ala (T/C)			
	TT *n* (%)	CT *n* (%)	CC *n* (%)	*p* ^a^
SCH male	160 (37.2)	143 (33.3)	127 (29.5)	0.001
Control male	250 (60.4)	116 (28.0)	48 (11.6)	
SCH female	48 (31.6)	64 (42.1)	40 (26.3)	0.001
Control female	117 (61.9)	52 (27.5)	20 (10.6)	

Abbreviations: *n*, number; SCH, schizophrenia.

^a^ p‐Values of the genotypic and allelic frequency distributions of polymorphisms between the groups were compared using the *χ* ^2^ test. In all cases, differences were considered significant at *p* < 0.05.

The analysis of TH levels, presented in Table 3, revealed significantly lower levels of fT3 and TSH in SCH patients compared with controls (*p* < 0.05 for both), while fT4 levels showed no significant difference (*p* > 0.05). When comparing hormone levels by sex within the SCH group, female patients exhibited significantly higher fT3 levels than male patients (*p* < 0.005), but no significant differences were observed for fT4 or TSH levels (Table 4).

**TABLE 3:** Thyroid hormone levels in study groups.

	SCH (*n* = 582)	Controls (*n* = 603)	*p*
fT3 levels pg/mL	3.88 ± 0.71	4.16 ± 0.69	**0.001**.
fT4 levels ng/dL	15.69 ± 1.64	15.62 ± 1.71	0.340
TSH levels IU/mL	1.99 ± 0.71	2.20 ± 0.78	**0.001**

*Note:* The analysis was done by the use of one‐way ANOVA with Tukey's post hoc analysis. Bold values (p < 0.05) were considered significant the differences between group.

Abbreviations: fT3, free triiodothyronine; fT4, free thyroxine; SCH, schizophrenia; TSH, thyroid‐stimulating hormone.

**TABLE 4:** Thyroid hormone levels with respect to gender in SCH patients.

	fT3 levels pg/mL		fT4 levels ng/dL		TSH levels IU/mL	*p*
Male (*n* = 430)	3.76 ± 1.18	**0.001**	15.67 ± 1.65	0.548	1.98 ± 0.68	0.401
Female (*n* = 152)	4.23 ± 0.71		15.76 ± 1.63		2.04 ± 0.77	

*Note:* The analysis was done by the use of one‐way ANOVA with Tukey's post hoc analysis. Bold values (p < 0.05) were considered significant the differences between group.

Abbreviations: fT3, free triiodothyronine; fT4, free thyroxine; SCH, schizophrenia; TSH, thyroid‐stimulating hormone.

**FIGURE 1:**
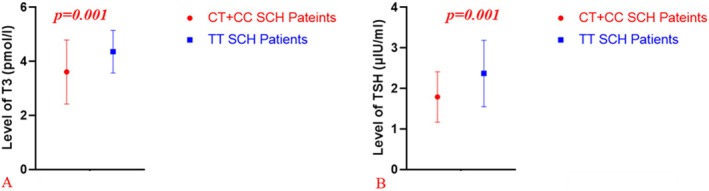
Associations between risk alleles carrying of Thr92Ala polymorphism and fT4 and TSH hormone levels in patients with SCH. fT3, free triiodothyronine; TSH, thyroid‐stimulating hormone. Statistical analysis was performed using the Student *t*‐test, and significance was determined at *p* < 0.05 in all cases.

The assessment of TH levels according to Thr92Ala and ORFa‐Gly3Asp genotypes indicated a significant association between the Thr92Ala polymorphism and reduced fT3 and TSH levels in SCH patients (*p* < 0.05), as shown in Figure 1 and Table 5. Individuals carrying the risk alleles associated with the Thr92Ala polymorphism exhibited lower fT3 and TSH levels compared to carriers of the wild‐type allele, while no significant difference was observed in fT4 levels. There was no significant association between the ORFa‐Gly3Asp polymorphism and TH levels. Additionally, the analysis of fT3, fT4 and TSH levels demonstrated no significant variations attributable to the influence of external therapeutic factors or specific polymorphic genotypes within the cohort. The assessment further indicated an absence of notable associations between thyroid hormone levels and the genetic variants studied, irrespective of clinical management approaches.

**TABLE 5:** Thyroid hormone levels with respect to Thr92Ala polymorphisms in SCH patients.

	fT3 levels pg/mL		fT4 levels ng/dL		TSH levels IU/mL	*p*
TT Genotype (*n* = 208)	4.36 ± 0.79	**0.001**	15.69 ± 1.63	0.915	2.19 ± 0.77	**0.018**
CT + CC Genotype (*n* = 374)	3.62 ± 1.1		15.70 ± 1.65		1.90 ± 0.66	

*Note:* The analysis was done by the use of one‐way ANOVA with Tukey's post hoc analysis. In all cases, differences were considered significant at *p* < 0.05. Bold values (*p* < 0.05) were considered significant the differences between group.

Abbreviations: fT3, free triiodothyronine; fT4, free thyroxine; SCH, schizophrenia; TSH, thyroid‐stimulating hormone.

## 4 Discussion

Genetic factors are increasingly recognised as contributors to the aetiology of psychiatric disorders. Polymorphisms within the DIO2 gene significantly affect various physiological processes and disease states [16]. Our study builds on the understanding of the genetic underpinnings of psychiatric disorders by examining the DIO2 gene polymorphisms' potential influence on SCH susceptibility. Variants in the DIO2 gene, which encodes an enzyme crucial for TH conversion, have been previously implicated in various physiological and neuropsychiatric conditions [22]. While extensive literature highlights the association between DIO2 variants and mood disorders like recurrent depressive disorder, studies linking these variants to schizophrenia remain sparse. In this study, we identified a significant association between the Thr92Ala polymorphism and SCH, suggesting that thyroid hormones may serve as biomarkers or modulators in SCH pathology and potentially influence symptomatology and medication responsiveness.

Many studies underscore the pivotal role of TH in the development of the brain and nervous system, highlighting its importance not only in adulthood but also during foetal development. Thyroid hormones, play a crucial role in brain development and the regulation of neurotransmitter systems, including dopaminergic, serotonergic and glutamatergic pathways, all of which are implicated in SCH [23]. Particularly triiodothyronine (T3), modulate neurotransmitter systems, including dopaminergic and glutamatergic pathways, which are implicated in SCH symptoms like psychosis [24]. Hypothyroidism, or insufficient thyroid hormone, has been associated with cognitive dysfunction and mood disturbances, which are commonly observed in SCH patients [25]. Dysregulated thyroid hormone signalling can impair neuroplasticity, affecting synaptic plasticity and memory processes, which are often disrupted in schizophrenia [23]. Also, suboptimal levels of TH exposure during foetal development have been implicated in the potential manifestation of mental retardation [26]. A very recent study investigated the association between Thr92Ala polymorphism in the DIO2 gene and SCH, along with its impact on TH levels. Statistical analyses revealed a higher frequency of the wild‐type TT genotype in controls compared to SCH cases, indicating a significant association between the Thr92Ala polymorphism and SCH. However, this polymorphism did not influence serum thyroid levels or the severity of schizophrenia [26]. In a cohort of 582 SCH patients and 603 controls, we identified a statistically significant association between the Thr92Ala polymorphism and SCH, suggesting that alterations in TH activity might be implicated in SCH pathology. Our findings align partially with prior studies, though a discrepancy was noted compared to Hanifi et al. who did not find an association between the Thr92Ala polymorphism and serum thyroid levels or SCH severity [27]. Subsequent investigations involving larger cohorts are imperative to elucidate whether the purported involvement of T3 dysregulation in SCH pathophysiology remains irrespective of the Thr92Ala genotype.

Animal models provide significant insights into the impact of DIO2 polymorphisms on neuropsychiatric outcomes. Mice carrying the Ala92‐DIO2 variant exhibit unfolded protein responses and hypothyroidism in various brain regions, reduced physical activity, increased sleep duration and impaired object memory. Importantly, administration of liothyronine, a synthetic T3 hormone, enhances T3 signalling in the brain and ameliorates cognitive function in those mice [28].

In SCH, a clinically significant correlation has been observed between fT3 levels and scores on the Drug‐Induced Extrapyramidal Symptoms Scale, suggesting that fT3 may serve as a predictive biomarker of treatment response [29, 30]. This finding emphasises the significant role of thyroid hormone activity in influencing neuropsychiatric symptomatology, especially in contexts where polymorphisms in DIO2 gene may be involved. The interplay between TH function and psychiatric conditions highlights the need for further research into the genetic and biological underpinnings of these disorders, potentially paving the way for novel therapeutic approaches.

In wild‐type mice, exposure to cold temperature resulted in approximately 4‐fold sympathetic activation of BAT, while DIO2 knockout mice exhibited a more pronounced sympathetic activation of BAT, approximately 10‐fold higher than that of their wild‐type counterparts [31]. Additionally, in Japanese quail, exposure to light‐induced DIO2 expression in the medial hypothalamus (MBH) indicates a potential regulatory role of light on thyroid hormone metabolism in the brain. Notably, mutations in the thyroid hormone transporter monocarboxylate transporter‐8 (MCT8) have been linked to psychomotor deficits in humans, while compensatory mechanisms involving DIO2 in mice mitigate these effects [32, 33]. Both DIO2 and MCT8 knockout mice display hypothyroidism in peripheral tissues and the brain, particularly in the striatum, leading to impaired motor skills [34]. Moreover, research demonstrated that triiodothyronine can enhance the clinical efficacy of antidepressants [35]. A study investigating the relationship between two TH activity genes, DIO1 and DIO2, and the antidepressant responses found that patients treated with sertraline combined with T3 (SERT‐T3) for 8 weeks showed significant interactions with the DIO1‐C785T genotype [34]. This suggests that this polymorphism may influence treatment efficacy, while DIO2 polymorphisms did not show a significant association with depression [36]. Interestingly, another study reported that polymorphism of the DIO2 gene is associated with recurrent depressive disorder [35]. Recent findings further highlight that SNPs in DIO1, DIO2 and SLCO1C1 showed high sensitivity and specificity in differentiating depressive disorder from BD, emphasising the role of genetic variations in TH pathways in delineating between different mood disorders [37].

Prior research has established a significant association between polymorphisms in the DIO2 gene and BD particularly in the Chinese population [9]. Specifically, statistical significance was found in allele and genotype frequencies for the ORFa‐Gly3Asp and Thr92Ala polymorphisms. In contrast, the present study presents different findings. Specifically, while both allele and genotype frequencies did not exhibit significant distribution at ORFa‐Gly3Asp, we observed closely similar results regarding the Thr92Ala polymorphism, which remained significant in the entire study population. Notably, our present study highlights the significant role of the Thr92Ala polymorphism, particularly among males, indicating its potential as a protective mutation in the Turkish population. Also, a recent study reported that DIO2 gene ORFa‐Gly3Asp and Thr92Ala polymorphisms play a role in the incidence of male patients with mild cognitive impairment disease (MCI) in Uygur population [38].

Two separate studies conducted in China have investigated the relationship between the DIO2 gene and mental retardation (MR), yielding significant findings [11, 27]. The first study found no significant association between the Thr92Ala polymorphism and MR; however, it did identify two SNPs, rs225012 and rs225010, which exhibited statistically significant correlations with MR [11]. These results established a positive link between DIO2 and MR, indicating that certain genetic variations may influence the risk of developing this condition. The second study, on the other hand, did not replicate the significance of these polymorphisms. Instead, a family‐based association study in the Qinba region of China revealed a significant association with different polymorphisms related to the DIO2 gene [27]. This discrepancy underscores the complexities in genetic associations and the influence of regional genetic backgrounds.

TH plays a vital role particularly in the brain and nervous system, especially during the foetal period, which is crucial for preventing MR [39]. Inadequate levels of thyroid hormones, essential for brain differentiation, neuronal migration, and myelination, may contribute to diseases related to DIO2 enzyme activity. These findings suggest that the DIO2 gene, particularly the Thr92Ala polymorphism, could be implicated in the susceptibility to SCH, thereby providing strong evidence for its role in the etiopathogenesis of sexually dimorphic SCH.

## 5 Conclusion

In conclusion, our study demonstrates a significant association between polymorphisms in the DIO2 gene and SCH, marking an important step in understanding the role of TH activity in this psychiatric disorder. The predictive value of the Thr92Ala polymorphism in the DIO2 gene suggests its potential as a biological marker for SCH. Our findings also highlight the intricate relationship between TH activity and cognitive function in SCH, with emerging evidence indicating a potential link between T3 levels and improved cognitive outcomes. Additionally, our study contributes to the growing body of literature on the physiological functions and clinical implications of DIO2 polymorphisms across various diseases. The observed associations between DIO2 polymorphisms and other psychiatric disorders, such as BD and recurrent depressive disorder, emphasise the multifaceted role of TH activity in mental health. Further research is needed to further elucidate the precise mechanisms by which DIO2 polymorphisms may influence TH activity and subsequently impact neurodevelopmental outcomes.

## Author Contributions

**Gokce Akan:** conceptualization (equal), data curation (equal), investigation (equal), methodology (equal), software (equal), visualization (equal), writing – original draft (equal), writing – review and editing (equal). **Ismael Chatita Adolf:** methodology (equal). **Adil Colak:** methodology (equal). **Seda Acar:** investigation (equal). **Fatih Oncu:** data curation (equal). **Dogan Yesilbursa:** data curation (equal). **Solmaz Turkcan:** data curation (equal). **Sema Bilgic Gazioglu:** project administration (equal), supervision (equal). **Fatmahan Atalar:** conceptualization (equal), data curation (equal), funding acquisition (equal), project administration (equal), supervision (equal), validation (equal), writing – review and editing (equal).

## Ethics Statement

This study was approved by the Research Ethics Committee of BRSHH according to the WMA Declaration of Helsinki.

## Consent

Written and signed informed consent was obtained from all subjects and/or their legal guardians.

## Conflicts of Interest

The authors declare no conflicts of interest.

## Data Availability

The data sets generated during the current study are available from the corresponding author upon reasonable request.
